# Supplementary material for: A fault-intrusion-tolerant system and deadline-aware algorithm for scheduling scientific workflow in the cloud
Source: PeerJ Comput Sci. 2021 Nov 2;7:e747. doi: 10.7717/peerj-cs.747 (PMC8576554; doi:10.7717/peerj-cs.747)
Supplement: Supplemental Information 6 [file peerj-cs-07-747-s006.zip › Myproject1.1/docs/org/cloudbus/cloudsim/core/predicates/class-use/Predicate.html]

Uses of Class org.cloudbus.cloudsim.core.predicates.Predicate


JavaScript is disabled on your browser.


- Overview
- Package
- Class
- Use
- Tree
- Deprecated
- Index
- Help

- Prev
- Next

- Frames
- No Frames

- All Classes

## Uses of Class org.cloudbus.cloudsim.core.predicates.Predicate

- Packages that use Predicate

  | Package | Description |
  | --- | --- |
  |  |  |
  | --- | --- |
  | org.cloudbus.cloudsim.core |  |
  | org.cloudbus.cloudsim.core.predicates |  |
- - ### Uses of Predicate in org.cloudbus.cloudsim.core

    Methods in org.cloudbus.cloudsim.core with parameters of type Predicate

    | Modifier and Type | Method and Description |
    | --- | --- |
    |  |  |
    | --- | --- |
    | `static SimEvent` | CloudSim.`cancel(int src, Predicate p)` Removes an event from the event queue. |
    | `static boolean` | CloudSim.`cancelAll(int src, Predicate p)` Removes all events that match a given predicate from the future event queue returns true if at least one event has been cancelled; false otherwise. |
    | `SimEvent` | SimEntity.`cancelEvent(Predicate p)` Cancel the first event matching a predicate waiting in the entity's future queue. |
    | `static SimEvent` | CloudSim.`findFirstDeferred(int src, Predicate p)` Find first deferred event matching a predicate. |
    | `SimEvent` | SimEntity.`getNextEvent(Predicate p)` Get the first event matching a predicate from the deferred queue, or if none match, wait for a matching event to arrive. |
    | `int` | SimEntity.`numEventsWaiting(Predicate p)` Count how many events matching a predicate are waiting in the entity's deferred queue. |
    | `static SimEvent` | CloudSim.`select(int src, Predicate p)` Selects an event matching a predicate. |
    | `SimEvent` | SimEntity.`selectEvent(Predicate p)` Extract the first event matching a predicate waiting in the entity's deferred queue. |
    | `static void` | CloudSim.`wait(int src, Predicate p)` Sets an entity's state to be waiting. |
    | `void` | SimEntity.`waitForEvent(Predicate p)` Wait for an event matching a specific predicate. |
    | `static int` | CloudSim.`waiting(int d, Predicate p)` Checks if events for a specific entity are present in the deferred event queue. |
  - ### Uses of Predicate in org.cloudbus.cloudsim.core.predicates

    Subclasses of Predicate in org.cloudbus.cloudsim.core.predicates

    | Modifier and Type | Class and Description |
    | --- | --- |
    |  |  |
    | --- | --- |
    | `class` | `PredicateAny` A predicate which will match any event on the deferred event queue. |
    | `class` | `PredicateFrom` A predicate which selects events from specific entities.  The idea of simulation predicates was copied from SimJava 2. |
    | `class` | `PredicateNone` A predicate which will **not** match any event on the deferred event queue. |
    | `class` | `PredicateNotFrom` A predicate which selects events that have not been sent by specific entities. |
    | `class` | `PredicateNotType` A predicate to select events that don't match specific tags. |
    | `class` | `PredicateType` A predicate to select events with specific tags. |

- Overview
- Package
- Class
- Use
- Tree
- Deprecated
- Index
- Help

- Prev
- Next

- Frames
- No Frames

- All Classes
